# Plant salinity stress, sensing, and its mitigation through WRKY

**DOI:** 10.3389/fpls.2023.1238507

**Published:** 2023-10-04

**Authors:** Gyanendra Kumar Rai, Sonal Mishra, Rekha Chouhan, Muntazir Mushtaq, Aksar Ali Chowdhary, Pradeep K. Rai, Ranjeet Ranjan Kumar, Pradeep Kumar, Francisco Perez-Alfocea, Giuseppe Colla, Mariateresa Cardarelli, Vikas Srivastava, Sumit G. Gandhi

**Affiliations:** ^1^ School of Biotechnology, Sher-e-Kashmir University of Agricultural Sciences and Technology of Jammu, Jammu, India; ^2^ Department of Botany, School of Life Sciences, Central University of Jammu, Samba, Jammu & Kashmir, India; ^3^ Infectious Diseases Division, Council of Scientific and Industrial Research (CSIR)-Indian Institute of Integrative Medicine (CSIR-IIIM), Jammu, India; ^4^ Advance Center for Horticulture Research, Udheywala, Sher-e-Kashmir University of Agricultural Sciences and Technology of Jammu, Jammu & Kashmir, India; ^5^ Division of Biochemistry, Indian Council of Agricultural Research (ICAR), Indian Agricultural Research Institute, New Delhi, India; ^6^ Division of Integrated Farming System, Central Arid Zone Research Institute, Indian Council of Agricultural Research (ICAR), Jodhpur, India; ^7^ Department of Nutrition, Centre for Applied Soil Science and Biology of the Segura (CEBAS), of the Spanish National Research Council (CSIC), Murcia, Spain; ^8^ Department of Agriculture and Forest Sciences, University of Tuscia, Viterbo, Italy

**Keywords:** abiotic stress, ABA signaling, transcription factors, food security, ROS, SOS pathway

## Abstract

Salinity or salt stress has deleterious effects on plant growth and development. It imposes osmotic, ionic, and secondary stresses, including oxidative stress on the plants and is responsible for the reduction of overall crop productivity and therefore challenges global food security. Plants respond to salinity, by triggering homoeostatic mechanisms that counter salt-triggered disturbances in the physiology and biochemistry of plants. This involves the activation of many signaling components such as SOS pathway, ABA pathway, and ROS and osmotic stress signaling. These biochemical responses are accompanied by transcriptional modulation of stress-responsive genes, which is mostly mediated by salt-induced transcription factor (TF) activity. Among the TFs, the multifaceted significance of WRKY proteins has been realized in many diverse avenues of plants’ life including regulation of plant stress response. Therefore, in this review, we aimed to highlight the significance of salinity in a global perspective, the mechanism of salt sensing in plants, and the contribution of WRKYs in the modulation of plants’ response to salinity stress. This review will be a substantial tool to investigate this problem in different perspectives, targeting WRKY and offering directions to better manage salinity stress in the field to ensure food security.

## 1 Introduction

Salinity stress is a foremost abiotic constraint that affects agricultural yields worldwide (Sanwal et al., 2022a). Nearly 20% (~310 million hectares) of the total irrigated land (1,500 million hectares) and 2% under dry land agriculture (~30 million hectares), across the world, have degraded due to high salts (Sanwal et al., 2022b). In India, it is estimated that ~10% of additional area is getting spoiled by salts every year and 2.1% (6.74 million ha) of total geographical area in India has already become salt affected (Kumar and Sharma, 2020). Plants’ reaction to environmental cues involve coordinated morphological, biochemical, and physiological responses, regulated by stress-responsive genes. Particularly with respect to high-saline conditions, genes related to synthesis and regulation of secondary metabolites, ion homeostasis, reactive oxygen species, salt overly sensitive (SOS) pathway, abscisic acid signaling, transcription factors (TFs), and mitogen-activated protein kinases (MAPK) are essential (Tuteja, 2007; Sytar et al., 2018). In fact, these mechanisms are also fundamental during chemical priming-based salt stress alleviation (Srivastava et al., 2021; Srivastava et al., 2022a; Mishra et al., 2023). Regulation of gene expression of associated pathways by TFs in response to various environmental triggers constitutes a basic regulatory mechanism of plants (Buscaill and Rivas, 2014). TFs comprise a significant portion of plant genome and are represented by many gene families such as NAC, AP2, MYB, and WRKY, which are reported to offer multifaceted impact on plant development and growth and regulate plants’ fitness against environmental constraints (Srivastava et al., 2022b; Chowdhary et al., 2023). WRKY proteins are among the important TFs involved in plants defense against several abiotic and biotic stimuli (Chen F. et al., 2017). These proteins are also known to be associated with different developmental and physiological processes in plants like seed and embryo development, trichome development, senescence, dormancy, and many metabolic pathways, and their role in mitigation of stress is widely studied (Eulgem et al., 2000; Pandey and Somssich, 2009; Chen et al., 2012; Yu et al., 2016a; Kang et al., 2021; Wani et al., 2021).

In plants, WRKY proteins constitute one of the biggest families of TFs, characterized by WRKYGQK DNA binding motif, which binds to W box (TTGACC/T) of the promoters (Eulgem et al., 2000; Rushton et al., 2010). Since their discovery in 1994, from sweet potato (Ishiguro and Nakamura, 1994, named as SPF1), WRKYs were thought to be exclusive to the plant kingdom. Later, Zhang and Wang in 2005 reported the presence of one copy of WRKY gene in *Giardia lamblia* (primitive protozoan), *Dictyosteliium discoideum* (slime mold), and *Chlamydomonas reinhaidtii* (green alga). With their origin in early eukaryotes, these genes have duplicated many times to evolve as an expanded super family of transcriptional regulators in land plants, viz., *Oryza sativa* L. ssp. *indica* (Ross et al., 2007), *Saccharum spontaneum* (Li et al., 2020b), *Medicago sativa* (Ma et al., 2021), and *Glycine max* (Yin et al., 2013), where their numbers reach hundreds. With this expansion in number, the WRKY superfamily has also been specified into three major sub-groups, namely, WRKY I, II, and III, based on the number of WRKY domains and Zn finger structure. The expansion of WRKY family in higher plants is due to segmental duplication events and subsequent divergent selection among the subgroups (Yin et al., 2013), which also diversify the functional prospects of WRKY protein family. *WRKY* genes are completely absent in kingdom Monera, Fungi, and Animalia (Zhang and Wang, 2005).

*WRKY* gene expression has been found to be induced in pathogenic conditions and other chemical and physical stresses (cold, heat, salinity, wounding, oxidative stress, and nutrition deficiency; Eulgem et al., 2000). Though the exact mechanisms of WRKY proteins are not well understood, it is reported that these factors repress or activate expression of other stress-responsive genes that ultimately confer protective effects. WRKY proteins are also known to regulate abscisic acid, ethylene, salicylic acid, and jasmonic acid signaling pathways, which mediate plant response to several stress conditions (Bakshi and Oelmüller, 2014) and are thus responsible for effective signal cross-talk and multifold regulations. Many investigations related to functional characterization of WRKYs have also suggested their contribution towards attainment of tolerance against abiotic stress like drought, heat, salt, and cold, and also offer resistance to pathogenic infections (Kumar et al., 2016; Gao et al., 2018; Shi et al., 2018; Wang et al., 2018; Gao et al., 2020; Yang et al., 2020; Kang et al., 2021). Moreover, WRKYs are also reported to regulate plant specialized metabolism (Mishra et al., 2013; Schluttenhofer and Yuan, 2015; Singh et al., 2017; Srivastava et al., 2017; Zhang et al., 2021).

Considering the significance of WRKY in plants’ life, many excellent reviews on general account of WRKY have been published (Eulgem et al., 2000; Rushton et al., 2010; Chen F. et al., 2017; Jiang et al., 2017; Wani et al., 2021), yet a judicial compilation of its role in individual stress is not much attempted. Nonetheless, several studies have been conducted in recent years to investigate its regulatory role in plant growth and development, and stress management, including salinity. The current review gives a comprehensive view on the WRKY-mediated plant response to salinity stress management and the associated mechanisms. The text discusses the impact of salinity stress and salt stress-related signaling mechanisms in plants, followed by a brief understanding of the WRKY gene family, their structure, and major classes in plant genome. Furthermore, it also highlights the various WRKY candidates involved in various stresses with a focus on salt stress tolerance and associated mechanism in plants.

## 2 Salinity stress and its impact on crop plants

The abiotic stresses decrease the yield, survival, and biomass of food crops by 70%, posing a serious risk to world food security (Ahmad et al., 2012; Parihar et al., 2015; Li et al., 2020a; Yoon et al., 2020; Ma et al., 2021). Salinity is one of the most serious constraints to crop development and productivity (Park et al., 2016). Among abiotic stress, the fraction of irrigated land affected by salt in different regions ranges from 9% to 34% with an average of 20% in the world (**Table 1**, cf. FAO-ITPS-GSP 2015). Salinity stress is the detrimental effect of excess elements like Na^+^ and Cl^−^ on plants (Parihar et al., 2015; Isayenkov and Maathuis, 2019). In addition, salinity is naturally complemented by secondary stresses like oxidative stress due to generation of ROS (Isayenkov, 2012; Mishra et al., 2017; Yang and Guo, 2018; Isayenkov and Maathuis, 2019). Based on its cause, salinity is categorized as primary or secondary (Kumar and Sharma, 2020). Primary (natural) salinity is developed due to the accumulation of salts during long-term natural processes (weathering of parent materials and inland oceanic salt deposition by wind/rain) in soil or groundwater. Contrary to this, secondary salinity involves various human interventions resulting in the alteration of soil–water equilibrium (Manchanda and Garg, 2008). Common examples of such human activities are deforestation, replacement of perennial crops with annual ones, irrigation with highly saline water, or inadequate drainage.

**Table 1:** Salt-affected soils in various regions of the world (cf. FAO-ITPS-GSP 2015).

Continent	Salt-affected area (mha)		
	Saline soils	Sodic soils	Total
Africa	122.9	86.7	209.6
Australasia	17.6	340.0	357.6
Mexico/Central America	2.0	–	2.0
North America	6.2	9.6	15.8
North and Central Asia	91.5	120.2	211.7
South America	69.5	59.8	129.3
South Asia	82.3	1.8	84.1
Southeast Asia	20.0	–	20.0
Total	412.0	618.1	1,030.1

**Figure 1:**
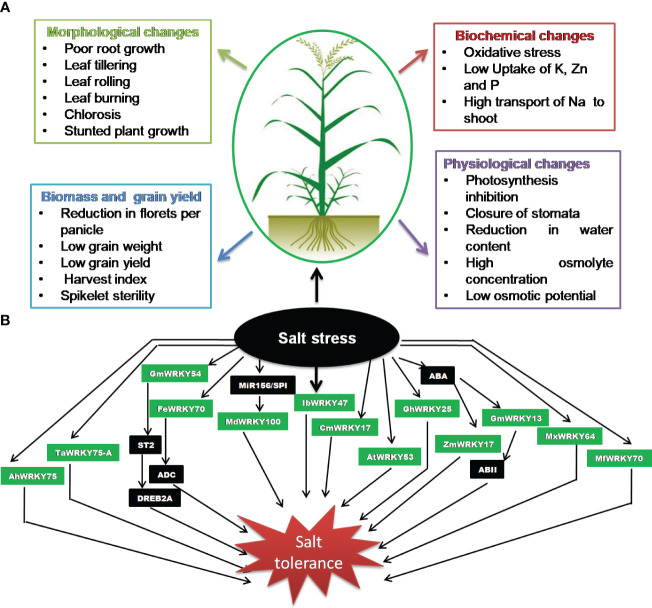
Salinity stress. **(A)** Impact on crop plants. **(B)** Significance of WRKY transcription factors (TFs).

Soil salinity is not a recent phenomenon; however, the issue has been accentuated as a result of agricultural activities such as intensive irrigation, poor water management, deforestation, and excessive use of pesticide and chemical fertilizers (Zhu, 2001; Tuteja, 2007; Gupta et al., 2022). It affects almost all the stages of growth and development in plants, from seed germination to blooming and seed maturation, thereby causing a significant loss in the crop yield (Singh et al., 2015; Srivastava et al., 2022a). Excess salt concentrations in the soil primarily affect ion balance in plants and create hyper osmotic stress and secondarily affect the accumulation of harmful ions, which results in poor or delayed germination and post-germination growth abnormalities (Majeed et al., 2019). It has been reported that a high Na^+^ concentration outside the plant cell has a negative impact on intracellular K^+^ influx, which is required for plant growth (Kumar and Sharma, 2020). Similarly, calcium and magnesium uptake by plants is also negatively impacted by high sodium content in saline soil. A disturbance in calcium uptake can lead to weakened cell walls, reduced enzyme activities, and altered signaling processes. Magnesium is critical for chlorophyll synthesis as well as production and transport of photoassimilates. During germination stage, salinity impairs the physiological function of seeds, which has a detrimental effect on seed germination and results in a general decrease in plant leaf area, biomass, yield, and root and shoot length (Zörb et al., 2019). Furthermore, it is known to cause various metabolic and physiological changes, depending on rigorousness and stress duration, and eventually reduces crop production (**Figure 1A**). The inhibitory effect of salinity on plant development involves reduction of water potential, disturbance of ion homeostasis, and associated cellular toxicity (Greenway and Munns, 1980; Isayenkov and Maathuis, 2019). In addition, it is also associated with numerous alterations in their physiology, such as hindering plant roots’ capacity to absorb water and essential minerals, reduction in the stomatal conductance, photosynthesis, and the inability for ROS detoxification, thereby inhibiting growth and development in plants (Abdallah et al., 2016; Ren et al., 2022; James et al., 2011; Gupta and Huang, 2014; Gulzar et al., 2019). Furthermore, the salinity-mediated oxidative stress causes accumulation of ROS such as superoxide anion, hydrogen peroxide, and the hydroxyl radicals, particularly in chloroplasts and mitochondria that damage cell membranes, proteins, lipids, and nucleic acids and may even lead to programmed cell death (Isayenkov, 2012; Mishra et al., 2017; Yang and Guo, 2018; Isayenkov and Maathuis, 2019).

## 3 Salt stress signaling pathway in plants

Plants differ widely in Na^+^ tolerance, and based on their capacity to tolerate salt stress, they are physiologically classified as glycophytes (low salinity tolerance) and halophytes (high salinity tolerance). The former (citrus, tomato, etc.) usually require fresh water and exhibit growth inhibition even under mild salinity. Citrus crops, therefore, showed signs of destruction and could not produce fruit and seeds even below 100 mM NaCl, whereas the halophytes can sustain and grow under elevated or high NaCl conditions (200 mM) (Flowers and Colmer, 2008; Flowers et al., 2010). Some plants such as *Atriplex, Rhizophora*, and *Suaeda* can even grow up to 1,000 mM NaCl (Ushakova et al., 2005; Park et al., 2016). Though the knowledge about sensor or receptor of Na^+^ is not known (Yang and Guo, 2018), it has been noticed that the ionic or osmotic stress may lead to increased cytosolic Ca^2+^ concentration (Kiegle et al., 2000; Choi et al., 2014). Furthermore, salinity treatment activates salt overly sensitive (SOS) pathway, abscisic acid (ABA) pathway, ROS signaling, and osmotic stress signaling (Yang and Guo, 2018).

One of the adaptive responses for cellular sustenance during salt stress is to retain ion homeostasis. This can be achieved by maintaining cytoplasmic K^+^/Na^+^ ratio by lowering Na^+^ and increasing K^+^ in the cytoplasm (Niu et al., 1995; Serrano et al., 1999), which involves Na^+^ uptake restriction, Na^+^ efflux enhancement, and Na^+^ compartmentalization in vacuole. Some of the specific transport system for Na^+^ and K^+^ uptake includes the low-affinity K^+^ channel (AKT1, *Arabidopsis* K^+^ Transporter1), the high-affinity K^+^ channel (HKT1, high-affinity K^+^ transporter 1), and the voltage-independent channel (Blumwald et al., 2000; Tuteja, 2007; Yang and Guo, 2018). Among these, HKT1 serves as a critical player in the improvement of tolerance to salinity by reducing Na^+^ accumulation in shoots, thereby avoiding Na^+^ toxicity in the leaves (Horie et al., 2005; Ren et al., 2005; Platten et al., 2006; Horie et al., 2009; Moller et al., 2009). Moreover, the contribution of Na^+^/Ca^2+^ exchanger-like proteins is also known to be prominent in ionic homeostasis (Mishra et al., 2021).

**Figure 2:**
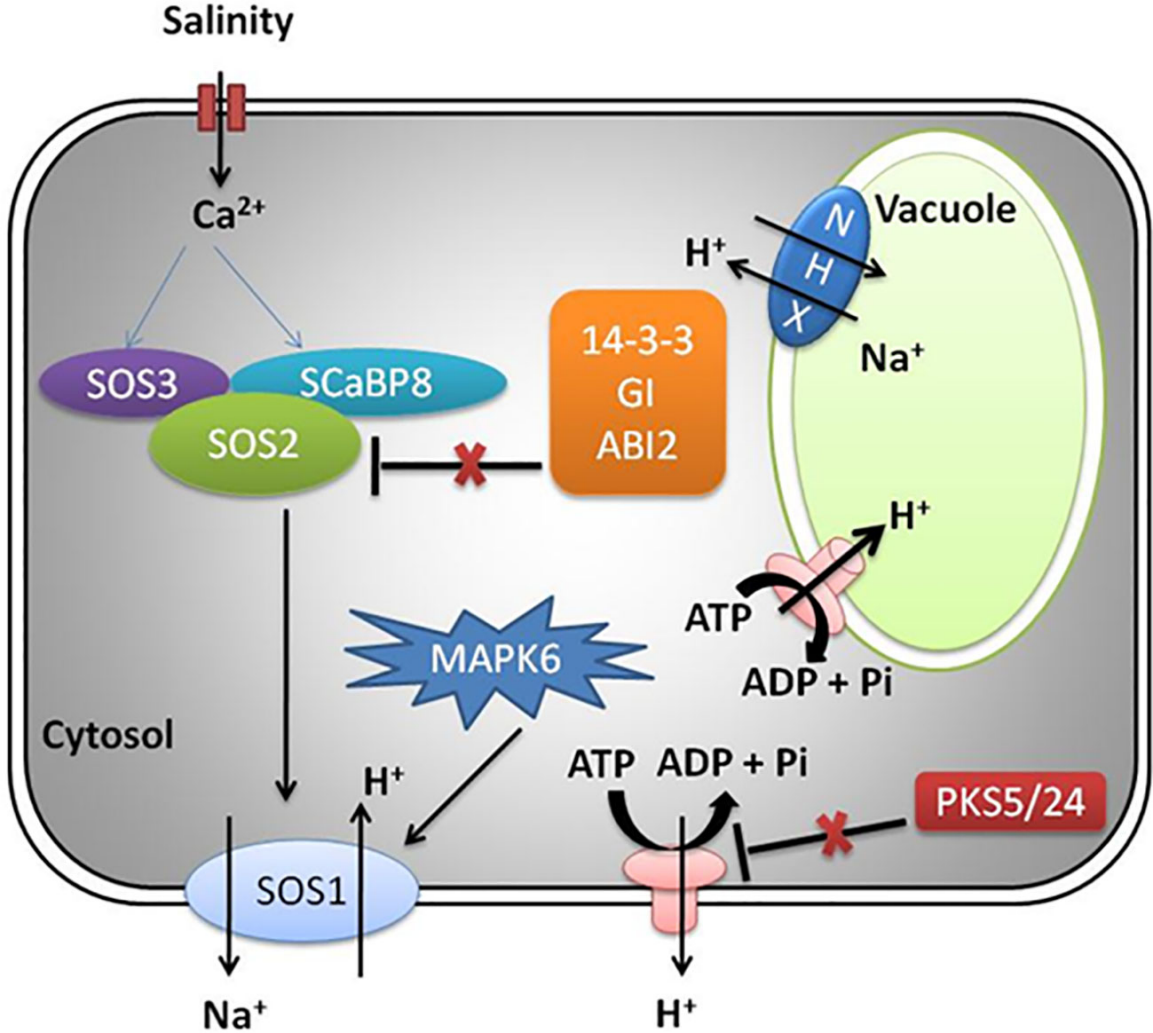
SOS pathway in plants under salinity stress.

The Na^+^ efflux mechanism is well characterized in *Arabidopsis* by genetic screening of SOS mutants exposed to salinity stress and reviewed in detail as presented in **Figure 2** (Yang and Guo, 2018). The SOS pathway exports Na^+^ ion from cells and involves activation of SOS2 (serine/threonine protein kinase) and SOS1 (Na^+^ antiporter) (Lin et al., 2009). The other players include helix E-loop-helix-F (EF-hand) calcium binding proteins (SOS3) and SCaBP8/CBL10, which recognizes high salt concentration and induction of cytosolic calcium signals (Liu and Zhu, 1998; Ishitani et al., 2000; Zhu, 2016). Under the influence of salt-induced cytoplasmic calcium induction, SOS3/SCaBP8 interact and induce SOS2 (Ishitani et al., 2000; Quan et al., 2007; Lin et al., 2009). The 14-3-3, GIGANTEA (GI), and ABA-INSENSITIVE 2 (ABI2) protein (phosphatase 2C) under non-saline (normal) conditions inhibit SOS pathway by interaction with SOS2, thereby repressing its kinase activity (Kim et al., 2013; Zhou et al., 2014; Yang and Guo, 2018). During salt stress, the 26S proteasome pathway degrades 14-3-3 and GI proteins. Additionally, PKS5 activity is also repressed, leading to normal functioning of PM H^+^-ATPase activity (Yang et al., 2010; Kim et al., 2013; Tan et al., 2016).

Na^+^ partitioning is also one of the adaptive responses that reduce cytoplasmic ionic toxicity, a mechanism conserved in glycophytes and halophytes (Blumwald et al., 2000; Hasegawa et al., 2000). Additionally, the abiotic stress including salinity leads to the generation of osmolytes, which can lower the water loss under short-term osmotic stress and enhances cell turgor during long-term osmotic stress (Apse and Blumwald, 2002). Furthermore, the osmotic stress also influences the regulation of enzymatic activities related to salt response.

The significance of ABA has also been observed in salinity stress. ABA induction under salt stress activates sucrose non-fermenting 1-related protein kinase 2 (SnRK2) kinase activities (Krzywinska et al., 2016); however, some evidence also indicated the ABA signaling-independent SnRK2 activation (Boudsocq et al., 2007; Zhang et al., 2011a; Zhang et al., 2016a). Additionally, it has been noticed that stimulation of salt stress led to the regulation of many stress-responsive genes, demonstrating correlation with osmotic stress. A study conducted by Sewelam et al. (2014) demonstrated induction of 932 genes under salt stress, out of which 435 overlap with transcripts induced by osmotic stress. Furthermore, 367 genes were found downregulated, in which 154 repressed genes were noted to overlap with osmotic stress (Sewelam et al., 2014).

The osmolytes can be grouped under several categories, viz., charged metabolites like proline, choline-O-sulfate, betaine, and glycine betaine; polyols like mannitol, glycerol, and myo-inositol; sugars such as fructose; complex sugars like fructans, raffinose, and trehalose; and ions such as K^+^ (Yang and Guo, 2018). Though these metabolites are accumulated in various plant species, few are specific to certain taxonomic categories. In addition, salt also induces the secondary stress response due to ROS generation (Ahmad and Prasad, 2011). ROS at low concentration functions as a signal; however, at high concentration, it has damaging effects over biomolecules (Miller et al., 2010; Gupta and Huang, 2014; Mishra et al., 2017). Therefore, tight regulation of ROS metabolism is a very important aspect for sustenance of normal plant growth under stress conditions. Furthermore, some small molecules act as signals, triggering downstream salt stress response (Yang and Guo, 2018), thereby improving salt tolerance, viz., proline (Khedr et al., 2003), carbon monoxide (Xie et al., 2008), phosphatidic acid (Yu et al., 2010), hydrogen sulfide (Christou et al., 2013; Srivastava et al., 2022a), γ-aminobutyric acid (Srivastava et al., 2021a), and melatonin (Liang et al., 2015; Wei et al., 2015; Mishra et al., 2023).

## 4 WRKY transcription factor family

TFs regulate expression of genes involved in diverse biological processes. More than 1,000 TF genes have been identified in angiosperms, which can be divided into 58 families depending on their DNA binding domains (Zhang et al., 2011b). WRKY is one of the most numerous TF families in plants involved in many signaling webs of several biological processes including specialized metabolism and stress tolerance (Rushton et al., 2010; Mishra et al., 2013; Kumar et al., 2016; Jiang et al., 2017). Being a TF, its predominant function is transcriptional modulation of genes by its repressor and activator (derepressor) activity. Since its initial reports (Ishiguro and Nakamura, 1994; Rushton et al., 1996), this protein family had been explored in several different plants that includes lower groups, eudicots, and monocots, and many excellent reviews are available mentioning its wide functional diversity (Eulgem et al., 2000; Rushton et al., 2010; Jiang et al., 2017). The investigations include model plants as well as several crops of high commercial significance (Chen F. et al., 2017). The development of sequencing technology has also triggered genome-wide investigation of imperative plant genes and many plant genomes have also been explored for the *WRKY* TFs (**Table 2**), which are mostly accompanied with expression study under diverse developmental, stress, and phyto-hormone treatment conditions (Kumar et al., 2016).

**Table 2:** Plant system explored for WRKY gene family survey and their representation in different sub-groups.

Plant systems	Number of WRKY TFs (ungrouped WRKYs)	Number of WRKYs in different subgroups								Reference
		Group I	Group II					Group III		
			IIa	IIb	IIc	IId	IIe	IIIa	IIIb	
*Actinidia deliciosa (Actinidia* spp.)	97	25	4	8	25	10	13	12		Jing and Liu (2018)
*Arabidopsis thaliana*	72	14	3	8	18	7	1	5	8	Li et al. (2014)
*Arachis duranensis*	75	16	4	10	18	7	7	13		Song et al. (2016)
*Arachis ipaensis*	77	14	4	10	18	7	9	15		Song et al. (2016)
*Artemisia annua*	122 (5)	26	8	10	18	16	10	29		De Paolis et al. (2020)
*Beta vulgaris* (Sugarbeet)	58	11	3	7	15	7	8	7		Wu et al. (2019)
*Brachypodium distachyon*	86 (2)	15	3	6	21	6	10	23		Tripathi et al. (2012)
*Brassica napus*	287 (5)	80	11	34	55	28	30	44		He et al. (2016b)
*Camellia sinensis* (Tea)	50	13	4	3	12	6	5	6		Wu et al. (2016)
*Cicer arietinum* (Chickpea)	78 (4)	13	5	11	16	6	12	11		Kumar et al. (2016)
*Coffea canephora*	49	10	3	6	15	6	4	5		Dong et al. (2019)
*Corchorus capsularis* (Jute)	43	9	2	7	7	6	6	6		Zhang et al. (2020)
*Cucumis sativus* (Cucumber*)*	62	11	8	9	12	7	8	7		Govardhana and Kumudini (2020)
*Dendrobium officinale* (Orchid)	63 (11)	14	4	3	9	6	6	10		He et al. (2017)
*Glycine max* (Soyabean)	188	32	14	33	42	21	20	26		Yu et al. (2016a)
*Glycyrrhiza glabra*	82	17	61					4		Goyal et al. (2020)
*Glycyrrhiza uralensis*	54	5	37					12		Goyal et al. (2020)
*Gossypium aridum*	109	17	7	15	30	15	13	12		Fan et al. (2015)
*Hevea brasiliensis* (Rubber)	81	16	6	11	17	9	8	12	2	Li et al. (2014)
*Hordeum vulgare* (Barley)	45	8	4	1	11	5	3	13		Mangelsen et al. (2008)
*Ipomoea batatas* (Sweet potato)	79	16	5	10	21	7	10	10		Qin et al. (2020)
*Malus domestica* (Apple)	127 (13)	23	8	27	13	13	16	14		Meng et al. (2016)
*Manihot esculenta* (Cassava)	85	17	5	14	20	8	9	12		Wei et al. (2016)
*Medicago sativa* (Alfalfa)	107	20	5	13	27	8	16	18		Mao et al. (2020)
*Medicago truncatula*	98 (7)	16	5	11	18	7	16	18		Kumar et al. (2016)
*Morus notabilis*	54 (1)	10	9	2	10	12	1	9		Baranwal et al. (2016)
*Oryza sativa*	98	17	4	8	16	7	1	8	26	Li et al. (2014)
*Pennisetum glaucum* (Pearl millet)	97	12	3	8	20	5	16	33		Chanwala et al. (2020)
*Phaseolus vulgaris* (Bean)	90 (2)	16	5	14	22	7	11	13		Wang et al. (2016)
*Populustrichocarpa*	100 (1)	22	5	9	27	13	13	10		Jiang et al. (2014)
*Prunuspersica* (Peach)	58	10	3	8	15	7	7	8		Chen et al. (2016)
*Ricinus communis* (Castor bean)	47	9	3	10	12	3	5	5		Li et al. (2012)
*Saccharum spontaneum*	154 (5)	17	6	12	40	11	12	51		Li et al, (2020b)
*Salix suchowensis*	85	19	4	8	23	13	11	7		Bi et al. (2016)
*Solanum lycopersicum*	81 (3)	15	5	8	16	6	17	11		Huang et al. (2012)
*Solanum tuberosum* (*Potato*)	79	13	5	6	18	7	16	14		Zhang et al. (2017)
*Sorghum bicolor* (Sorghum)	94 (2)	11	4	8	20	6	12	31		Baillo et al. (2020)
*Theobroma cacao*	61 (3)	10	3	8	17	6	6	8		Silva Monteiro de Almeida et al. (2017)
*Triticum aestivum*	171	30	11	7	50	17	10	45		Ning et al. (2017)
*Vitis vinifera* (Grapevine)	59 (2)	12	3	8	16	6	6	6		Wang et al. (2014)
*Zea mays (Maize)*	136	27	7	11	29	14	17	31		Wei et al. (2012)
*Ziziphus jujuba* (Chinese jujube)	61 in Junzao variety	10	3	10	14	5	8	11		Chen et al. (2019)
	52 in Dongzao variety	10	2	8	12	3	5	12		

Currently, the scope of the WRKY family has achieved a broader perspective. In reference to functional diversity, the WRKYs are associated with numerous functions in plants including germination, growth and development, flowering, senescence, carbohydrate synthesis, and secondary metabolite synthesis (Yu et al., 2016b; Jiang et al., 2017; Yu et al., 2018). In numerous studies, it has been reported that WRKY TFs enhance tolerance to salinity stress (Lv et al., 2020; Zhu et al., 2020), drought stress (He et al., 2016a; Wang et al., 2018), heat stress (He et al., 2016a; Wang et al., 2018), chilling stress (Zhang et al., 2016b), heavy metal stress (Sheng et al., 2019), and biotic stress (Cheng and Wang, 2014; Bai et al., 2018) in plants.

Structurally, WRKY proteins consist of 60-amino-acid-long highly conserved WRKY domains. These WRKY domains are made up of four β-strand structures and a C-terminal zinc binding Cystine/Histidine finger motif (Eulgem, 2006; Rushton et al., 2010). The β-strand at the N-terminal contains a conserved stretch of seven amino acids also referred to as “WRKY Signature”, usually composed of “WRKYGQK”, while some WRKY variants, viz., WRKYGEK, WRKYGKK, WRICGQK, WRMCGQK, WKKYGQK, WIKYGQK, WKRYGQK, WSKYEQK, WRKYSEK, WRRYGQK, WSKYGOK, WVKYGQK, WRICGQK, and WRMCGQK, have also been reported in this family (Jiang et al., 2017). The hepta-peptide stretch is considered essential for WRKY binding to the gene promoters [at specific location referred as W-Box–(T)TGAC(C/T)], and hence, alterations in this pattern could lead to changes in their DNA binding ability (Chen F. et al., 2017). W-box components are typical in plant genomes and are made up of a conserved GAC core, a downstream pyrimidine (C/T) residue, and an upstream thymine residue. Although the core aids in WRKY binding, the neighboring residues provide specificity for recognition of a given W-box by a specific factor. For effective binding of WRKYs, more than one W-box can occur in proximity. Certain WRKY are also known to regulate gene expression by binding to elements other than W-box, which includes WT-box (GGACTTTC), WK-box (TTTTCCAC), PRE4-element (TGCGCTT), and SURE-element (TAAAGATTACTAATAGGAA) (Phukan et al., 2016; Chen F. et al., 2017). Other domains also exist among some members of WRKY, including nuclear localization signals (NLS), calmodulin binding sites (CBS), proline-rich region, nucleotide-binding site, leucine-rich repeat, toll interleukin-1 receptor (TIR), NAC (NAM, ATAF1/2 and CUC2) domain, SQUAMOSA promoter binding protein (SBP) domain, ubiquitin-like protease domain, paired amphipathic helix (PAH) domain, ATP-grasp, and other structures. These systems may provide additional functional benefits to WRKY TFs (Eulgem, 2006; Chen F. et al., 2017; Jiang et al., 2017).

The WRKY TFs have been classified into three groups (**Table 2**) depending on number of WRKY domains (WDs) and pattern of Zn finger motifs. Group I is composed of two WDs with C2H2-type zinc-finger motif, whereas group II has one WD with C2H2-type zinc-finger motif. Group III also possesses single WD like group II, but possesses C2HC-type zinc-finger motif (Kumar et al., 2016). Furthermore, phylogenetic analysis, conserved domain structures, and intron position of the WDs demonstrated further sub-grouping of WRKY TFs (Eulgem et al., 2000; Zhang and Wang, 2005; Kumar et al., 2016). Group II WRKYs are subdivided into five subgroups, namely, IIa, IIb, IIc, IId, and IIe. Group III WRKYs are also composed of two subgroups, namely, IIIa and IIIb (Wu et al., 2005; Zhu et al., 2013). The WRKY domain at the C-terminus of group I proteins is thought to be necessary for DNA binding activity and exhibits similarity to the WRKY domains of group II and group III proteins. WRKY TFs are further classified into two types: R-type and V-type *WRKYs*, based on the position of intron. The R-type WRKY has a splicing site between the first and second Gs of the AGG codon (arginine), while the V-type WRKY has a splicing site after the valine codon, which is located after the sixth amino acid from the second cysteine residue of the zinc-finger motif (Jiang et al., 2017).

## 5 WRKYs mediated transcriptional modulation, its interacting partners, and significance under a stressed environment

WRKYs function as either activators or repressors in a variety of molecular processes. They act in an auto-regulated or cross-regulated manner by interacting with other WRKY members or different proteins such as MAP kinases, calmodulin, histone deacetylases, 14-3-3 proteins, and VQ proteins (Rushton et al., 2010; Chi et al., 2013; Phukan et al., 2016). Sometimes, a single WRKY may exhibit several responses, while several WRKYs may also work together to mediate a particular response (Phukan et al., 2016).

Various transcriptional, post-transcriptional, post-translational, and proteasome-mediated mechanisms are known for regulating expression and downstream activation of WRKY in normal and stressed conditions. A zinc-finger protein, Zat12, induced by various abiotic stimuli (salinity, drought, and wounding) was reported to regulate the expression of *AtWRKY25* (Mittler et al., 2006). Certain MYB TFs also regulate the expression of *WRKYs* (Ishida et al., 2007). Transcription of many *WRKYs* is also regulated by signal molecules. PTI [pathogen-associated molecular patterns (PAMPs)-triggered immunity]- and ETI (effector-triggered immunity)-mediated activation of WRKYs has been observed under several biotic stresses. NaCl treatment induced the expression of *WRKY25* and *WRKY33* in *A. thaliana*, and their overexpression increased tolerance to salinity stress (Jiang and Deyholos, 2009). Similarly, overexpression of *GmWRKY54* in *A. thaliana* increased the plant’s tolerance to salt stress. Salt stress also led to accumulation of OsWRKY54 in rice, which, in turn, regulated the expression of *OsHKT1;5* by binding to the W-box motif in its promoter. Extensive similarities and cross-talk exist between salinity and drought stress responses in plants (Golldack et al., 2014). WRKY46, WRKY54, and WRKY70 together interact with BES1 to regulate brassinosteroid-mediated drought response (Chen J. et al., 2017). DREB TFs are considered as master regulators in drought response. Regulation of DREBs by TaWRKY19 (Niu et al., 2012) and GhWRKY59 (Jin et al., 2017) is another example of crosstalk between TFs mediating salt response. On the other hand, there are also examples where WRKYs function differently in salt and drought stress. For instance, overexpression of *GhWRKY25* in *Nicotiana benthamiana* increases salinity tolerance but negatively impacted drought tolerance and sensitivity to fungal pathogen. Expression of *WRKY* was also reported to be controlled by miRNAs at the post-transcriptional level (Phukan et al., 2016). Interactions of histone deactelyases (HDAC), histone demethylase, and histone methyl transferases with *WRKY* revealed the non-genetic regulation of WRKYs in plants (Chi et al., 2013; Phukan et al., 2016). Histone deactylase-19 removes acetyl groups from histone tails and downregulates the expression of *AtWRKY38* and *AtWRKY62* (Kim et al., 2008). The linker histone H1 MaHIS1 interacts with MaWRKY1 and functionally coordinates to influence stress responses and ripening in banana fruit (Wang et al., 2012b). Flowering Locus D (FLD) brings about histone modifications of *WRKY* 29 and *WRKY6* gene promoters and, thus, epigenetically regulates their SAR (systemic-acquired resistance)-induced expression (Singh et al., 2014). Chloroplast- and mitochondria-mediated retrograde inter-organelle signaling to the nucleus regulates several *WRKY* factors (Hammargren et al., 2008; Shang et al., 2010). Furthermore, phosphorylation by kinases is also known to modulate the expression and functioning of *WRKY* TFs. MAPK regulates the expression of *OsWRKY45* and provides resistance to various pathogenic infections in rice. Responses to bacterial and fungal infections are also modulated by *AtWRKY22* and *AtWRKY29* through the MAPK pathway (Göhre et al., 2012). WRKYs in tobacco interact with MAPK cascade pathways in plant defense against whiteflies (Yao et al., 2021). Proteasome-mediated degradation also maintains the level of *WRKYs* under various stressed and non-stressed conditions. UPS (ubiquitin proteasome system) is known to degrade *OsWRKY45* at normal un-diseased state in plants, whereas the pathogenic invasions inhibit proteasomes and accumulate *OsWRKY45* (Matsushita et al., 2013; Phukan et al., 2016).

## 6 *WRKY*s and crop improvement for salt tolerance involve multiple responses

WRKYs play promising roles during plant signaling and are extensively reported for their contributions in abiotic and biotic stress (Li et al., 2020a; Wani et al., 2021). Nevertheless, current advances do divulge the vast significance of WRKY proteins for regulation of plant abiotic stress tolerance (Huang and Amee, 2021; Xiang et al., 2021). Researchers have employed specific WRKY TFs to create transgenics with improved stress tolerance traits (**Table 3**), because of their regulatory effects on stress-responsive genes clusters (Banerjee and Roychoudhury, 2015). Understanding of the signaling cascades that lead to the activation and interaction of the WRKY proteins with other signaling proteins, and the regulation of downstream target genes are crucial in the choice of WRKY genes for engineering stress tolerance in plants.

**Table 3:** Functional characterization of WRKYs towards salt stress and associated mechanism.

Plant system	Type of WRKY protein	Response to salinity	Biochemical and physiological changes	References
*Arabidopsis thaliana*	AtWRKY33	Enhances salinity tolerance in transgenic *Arabidopsis thaliana*	Improved stress tolerance via increased seedling length, reduced oxidative stress, as well as by preventing leaf chlorosis.	Jiang and Deyholos (2009)
*Brassica campestris*	BcWRKY46	Enhanced salinity tolerance in transgenic *Nicotiana tabacum*	Enhanced stress tolerance by increasing seed germination, mediated signal transduction, as well as by activating the expression of osmotic stress genes.	Wang et al. (2012a)
*Dendronthemagrandiform*	DgWRKY5	Improved salinity tolerance in transgenic *Dendronthema grandiform.*	Improved stress tolerance via improvements to a number of growth characteristics, including root length, chlorophyll content, fresh weight, and leaf gas exchange parameters as well as by reduced oxidative stress via upregulating the activity of antioxidant enzymes as well as the expression of genes associated with stress.	Liang et al. (2017)
*Fagopyrumtataricum*	FtWRKY46	Enhanced salinity stress tolerance in transgenic *Arabidopsis thaliana*	Enhanced stress tolerance by modulating the ROS clearance as well as the expression of stress-responsive genes.	Lv et al. (2020)
*Glycine max*	GmWRKY49	Improved salinity stress tolerance in transgenic *Glycine max and Arabidopsis thaliana*	Enhanced stress tolerance by improving several growth parameters like germination rate, root length, survival rate, and rosette diameter by reducing oxidative stress as well by regulating downstream stress-responsive genes.	Xu et al. (2018)
*Glycine max*	GmWRKY12	Confers salt tolerance in transgenic *Glycine max*	It confers salt stress tolerance by lowering oxidative stress, as evidenced by higher proline content and lower malondialdehyde (MDA) content in transgenic lines	Shi et al. (2018)
*Glycine max*	GmWRKY54	Improved salinity stress tolerance in transgenic *Glycine max*	Improved stress tolerance via regulated DREB2A and STZ/Zat10.	Zhou et al. (2008)
*Gossypium hirsutum*	GhWRKY68	Reduced salinity tolerance in transgenic *Gossypium hirsutum*	Sensitive to oxidative stress.	Jia et al. (2015)
*Gossypium hirsutum*	GhWRKY17	Reduced salt tolerance	The transgenic *Nicotiana benthamiana* overexpressing Gh WRKY17 exhibited impaired stomatal closer and also modulate the antioxidant defense mechanism.	Yan et al. (2014)
*Ipomoea batatas* L.	IbWRKY2	Increased salinity stress tolerance in transgenic *Arabidopsis thaliana*	Increased stress tolerance via reduced oxidative stress by increasing gene expression, associated with the ABA signaling pathway, proline biosynthesis, and ROS-scavenging system	Zhu et al. (2020)
*Jatropha curca*	JcWRKY	Improved salt stress tolerance in transgenic *Nicotianata tabacum* L.	Improved stress tolerance via improvement in several growth parameters such as increasing germination potential, membrane stability, as well as by reducing oxidative stress via improved activity of antioxidant enzymes.	Agarwal et al. (2016)
*Malus baccata* (L.) Borkh	MbWRKY5	Increases salinity tolerance in transgenic *N. tabacum* var. Xanthi	Increased stress tolerance by reducing oxidative stress via improving activity of antioxidant enzymes as well as increased expression of stress-responsive genes.	Han et al. (2018)
*Malus domestica*	MdWRKY30	Improved salinity stress tolerance in transgenic *Arabidopsis thaliana*.	Improved stress tolerance via transcriptional regulation of stress-related genes.	Dong et al. (2020)
*Malus domestica*	MdWRKY100	Enhances salinity tolerance in transgenic *Malus domestica*	Improved stress tolerance via reduced oxidative stress.	Ma et al. (2021)
*Malus xiaojinensis*	*MxWRKY55*	Improved salinity tolerance in transgenic *Arabidopsis thaliana*	It enhances tolerance to stress by increasing proline and chlorophyll content. Improving the antioxidant defense system, which reduced malondialdehyde content	Han et al. (2020)
*Oryza sativa*	*OsWRKY72*	Increased susceptibility to salinity stress in transgenic *Arabidopsis thaliana* and salt sensitivity in *Oryza sativa*.	Exogenous application of ABA and NaCl induced *OsWRKY72* expression in rice under salinity stress and improved the salt tolerance in rice by upregulation of *OsWRKY72*	Song et al. (2010)
*Pennisetum glaucum*	PgWRKY33/62	It enhances salt tolerance in pearl millet	PgWRKY62 was significantly unregulated in salt-treated pearl millet plants. Differential expression pattern in response to salinity stress in various tissue such as leaf, stem, and root.	Chanwala et al. (2020)
*Phyllostachys edulis*	PeWRKY83	Enhanced salinity stress tolerance in transgenic *Arabidopsis thaliana*	It improves stress tolerance by regulating the stress-induced synthesis of ABA.	Wu et al. (2017)
*Populus alba*	PagWRKY75	Negatively regulate salt stress in *Populus alba*	PagWRKY75 reduces the ROS scavenging ability and proline accumulation under various stresses, and positively regulates the water loss rate of leaves. Thus, PagWRKY75 can negatively regulate salt and osmotic tolerance by altering various physiological processes.	Zhao et al. (2019)
*Solanum lycopersicum* L.	SlWRKY8	Mediates salt stress tolerance in transgenic *S*. *lycopersicum* L.	Mediate salinity stress tolerance by reducing oxidative stress via increased activity of antioxidant enzymes.	Gao et al. (2020)
*Triticum aestivum* L.	TaWRKY2/19	Improved salinity tolerance in transgenic wheat	Improved stress tolerance by regulating downstream stress-responsive genes.	Niu et al. (2012)
*Triticum aestivum* L.	*TaWRKY93*	Enhanced salinity stress tolerance in transgenic *Arabidopsis thaliana*	It enhances salinity tolerance by enhancing osmotic adjustment, and regulates transcription of stress-responsive genes.	Qin et al. (2015)
*Vitis pseudoreticulata*	*VpWRKY3*	Improves salinity tolerance in transgenic *N. tabacum*	*VpWRKY3* is involved in abscisic acid signal pathway.	Zhu et al. (2012)
*Vitis vinifera*	*VvWRKY30*	Improves salinity tolerance in transgenic *Arabidopsis thaliana*	Controlling the scavenging of reactive oxygen species as well as accumulating osmoprotectants.	Zhu et al. (2019)
*Zea mays*	*ZmWRKY17*	Increased susceptibility to salinity stress in transgenic *A. thaliana*	Increased susceptibility to salinity stress via regulation of stress-responsive genes.	Cai et al. (2017)

Salinity stress is a key abiotic stress that affects agricultural productivity, mostly in semi-arid and arid areas. WRKYs are known to play a critical role in the regulation of plant salt stress responses (**Figure 1B**; **Table 3**). WRKY has been observed as both a positive (Han et al., 2021; Xiang et al., 2021; Ye et al., 2021; Zhu et al., 2021) and a negative regulator (Huang and Amee, 2021) for salinity stress. In a study, 47 *WRKY* genes were reported to respond to salinity stress in wheat (Hassan et al., 2019), which demonstrated the significance of WRKY during salinity stress. The STZ (zinc finger protein STZ/ZAT10) protein associated to ZPT2 (zinc finger protein) is known for downregulating the deactivation of other TFs and, therefore, functions as an inhibitor of transcription. Zhou et al. (2008) reported that the *STZ* expression is inhibited by GmWRKY54 in *G. max*, thus inducing response to salt stress via the positive regulation of DREB2A-mediated pathway (Zhou et al., 2008). In another study, Gong et al. (2015) demonstrated that FcWRKY70 is involved in upregulating expression of *arginine decarboxylase* (*ADC*), resulting in plant salinity tolerance. The *miR156/SPL* is involved in modulation of tolerance to salinity stress by upregulating *MdWRKY100* in *Malus domestica* (Ma et al., 2021). The SbWRKY50 directly binds to *SOS1* and *HKT1* promoter and participated in plant salt response by regulating ion homeostasis in *Sorghum bicolor* (Song et al., 2020). The salt tolerance in transgenic *Arabidopsis*-overexpressing peanut *AhWRKY75* (Zhu et al., 2021) involved the upregulation of genes associated with ROS scavenging activity and improved antioxidant system (SOD, POD, and catalase). Furthermore, the significantly lower accumulation of malondialdehyde and superoxide anion content was also noticed in transgenic plants (Zhu et al., 2021). Similar observation was also noticed in transgenic *Arabidopsis* overexpressing *Myrothamnus flabellifolia MfWRKY70*-mediated salt tolerance (Xiang et al., 2021). The transgenic plants demonstrated the positive regulation of stress-associated genes such as *P5CS, NCED3*, and *RD29A.*

The salinity (and drought) tolerance in the ectopically expressed *TaWRKY75-A* in *Arabidopsis* integrated jasmonic acid biosynthetic pathways (Ye et al., 2021). In contrast, an increased expression level of *GhWRKY25* increases the salinity stress tolerance in upland cotton, whereas transgenic tobacco plant showed comparatively lower drought stress tolerance, signifying that the WRKY exhibited different regulatory effects in response to diverse stress conditions (Liu et al., 2016). Shen et al. (2015) revealed that the antioxidant enzyme activity is enhanced during salt-induced H_2_O_2_ and cytosolic Ca^2+^ stimulation in *Populus euphratica*, thus improving salt stress tolerance. Salinity stress response has been shown to be largely related to ABA-induced *WRKY* gene expression (Li et al., 2020a). Various reports have demonstrated that ABA and NaCl when applied exogenously can also induce *WRKY* expression like *AtWRKY25* and *AtWRKY33* in *Arabidopsis* (Jiang and Deyholos, 2009), *OsWRKY72* in rice (Song et al., 2010), *GbWRKY1* in *Gossypium barbadense* (Luo et al., 2020), and *VpWRKY3* (Zhu et al., 2012) and *VpWRKY1/2* (Li et al., 2010) in grape. Functional studies of WRKYs towards salt stress tolerance have been compiled in **Table 3**, which also explains the pathways regulated during WRKY-mediated tolerance to the salinity stress.

Additionally, WRKYs are also known as negative regulators of salt stress tolerance trait in plants (Zhou et al., 2008; Huang and Amee, 2021). The inhibition of salt stress tolerance via regulation of the DNA binding and transcriptional activity of WRKY53 was reported by *Arabidopsis* RPD3-like histone deacetylase HDA9 (Zheng et al., 2020). Li et al. (2015) reported overexpression of *Chrysanthemum CmWRKY17* in *Arabidopsis*, which resulted in higher sensitivity towards salt stress. The study reported that stress resistance-related genes in wild-type plants showed higher expression against stress compared to transgenic *Arabidopsis*, demonstrating that *CmWRKY17* may be implicated in negative regulation of salinity stress in *Chrysanthemum* (Li et al., 2015). Similarly, salinity sensitivity was also observed in *CdWRKY50* overexpressing *Arabidopsis.* The CdWRKY50 can also bind to the *AtDREB2A* promoter, thereby regulating its expression (Huang and Amee, 2021). In *G. max*, ABI1 could be the downstream target gene of GmWRKY13. Transgenic studies in *Arabidopsis* exhibited that the overexpression of *GmWRKY13* enhanced *ABI1* expression; however, plants were found to be less tolerant to salt stress (Zhou et al., 2008). Overexpression of *ZmWRKY17* in *Arabidopsis* demonstrated an inhibitory result on exogenous ABA treatment, ensuing comparatively lower tolerance to high salinity (Cai et al., 2017).

Although the literature strongly supported this function of WRKY in salinity stress, there are certain missing links that need reasonable research, viz., How does salt stress cause WRKY induction? Is this generalized or specific to plant/members of WRKY gene family? Does post-translational modification of WRKY impact its functionality during salt stress? Does the homo- and heterodimerization of WRKY influence its behavior during salinity? How does the WRKY-mediated metabolite regulation influence its role in salt stress mitigation?

## 7 Conclusion and future directions

The ultimate solution to ensure crop production potential is to incorporate tolerance traits into the plants. The significant impact of salinity stress over crop production is an urgent challenge to ensure sustainable crop production to feed the global population. Salinity stress significantly deteriorates the crop production potential throughout the globe, due to its larger effect on plant physiology and biochemistry, thus ultimately leading to significant agricultural loss. Plants differ significantly in terms of their tolerance to salinity and have the capacity to sense this stress through the SOS pathway, which involves many candidate proteins. Among several tolerance mechanisms to address salinity stress-mediated crop loss, the utility of TF-mediated tolerance is well documented. Being one of the major TF families, WRKY plays a significant role in plants at several avenues including stress tolerance (plant fitness to environmental constraints). Over the years, scientists have revealed that WRKY TFs not only contribute to growth and development in plants, but also exhibit complex regulatory networks and mechanism implicated in various stresses. Since crops generally face different stresses and WRKYs play crucial roles during stress response, further detailed studies on *WRKY* genes are needed to specify their unique functions. So far, characterization of WRKY is considered, and many plants have been established as a model to support the significance of WRKY in salt tolerance. Furthermore, the underlying mechanism is also explored at few instances (**Table 3**) but broader validation is needed. In addition, genomics has facilitated exploration of this protein family in many crops and newer studies are continuously enriching this data. Furthermore, such investigation offers a broader perspective as the researcher can individually target most promising WRKYs out of close putative candidates. Moreover, earlier research work over *WRKY* gene functions was mostly focused on transcriptomics and functional predictions, while further applications of genetic confirmation integrated with novel tools help to speed up the research regarding studies related to WRKY neo-functionalization. Further characterization of the downstream genes that are regulated through WRKY is still a challenge. Such research explorations will help to elucidate the regulatory networks involved in stress response in plants. Furthermore, non-coding RNAs (ncRNA) and epigenetic modifications entailed in the *WRKY* TFs regulation must be investigated in future research. By integrating multiomics methods such as genomics, transcriptomics, proteomics, and metabolomics, TFs have been investigated and further modified utilizing genome editing tools such as CRISPR/Cas systems to improve plant tolerance to various abiotic stresses such as salt stress. In-depth studies of TFs will possibly enhance our ability to improve the stress tolerance in crop plants to achieve food security at the global level. Finally, using WRKY TFs to monitor stress-tolerant plant cultivars and enhance stress resistance in plants will considerably help to improve quality and yield in the perspective of climate change and food security.

## Author contributions

GR, VS, MC, and SG: Framing the concept and writing; SM, RC, MC, and MM: Manuscript writing; AC, RK, PK, and PR: prepared the figures and tables; FP-A, RK, MC, and PK: Manuscript correction; SG, MC, GC, FP-A, PK, and VS: modified and edited the manuscript. All authors contributed to the article and approved the submitted version.
